# Serotonergic neurotransmission mediated cognitive dysfunction in two mouse models of sepsis‐associated encephalopathy

**DOI:** 10.1111/cns.14655

**Published:** 2024-03-03

**Authors:** Chen Zhang, Fafa Tian, Jing Peng, Xia Wang, Jingchen Li, Lina Zhang, Zheren Tan

**Affiliations:** ^1^ Department of Pediatrics, Xiangya Hospital Central South University Changsha China; ^2^ Department of Neurology, Xiangya Hospital Central South University Changsha China; ^3^ Bioinformatics Center & National Clinical Research Centre for Geriatric Disorders & Department of Geriatrics, Xiangya Hospital Central South University Changsha Human China; ^4^ Department of Critical Care Medicine, Xiangya Hospital Central South University Changsha China; ^5^ National Clinical Research Center for Geriatric Disorders Changsha Hunan China; ^6^ Hunan Provincial Clinical Research Center for Critical Care Medicine Changsha Hunan China

**Keywords:** cognitive dysfunction, sepsis‐associated encephalopathy, serotonergic neurotransmission, serotonin, therapeutics

## Abstract

**Background:**

Patients with sepsis‐associated encephalopathy (SAE) often exhibit cognitive impairments. Despite this, the underlying mechanisms of SAE remain largely unexplored. Here, we explored the role of serotonergic neurotransmission in cognitive dysfunction of two mouse models of SAE.

**Methods:**

The mouse models of SAE were established by injection of lipopolysaccharide (LPS, 10 mg/kg, intraperitoneal) and cecal ligation puncture (CLP) respectively. Barnes maze, new object recognition test and open field test were used to evaluate the effects of fluoxetine (selective serotonin reuptake inhibitor) and cyproheptadine (nonselective 5‐HT_2_ receptor antagonist) on cognition and motor activity of mice. Additionally, WAY100635 (5‐HT_1A_ receptor antagonist) was co‐administered with fluoxetine to explore the mechanism underlying effect of fluoxetine on cognitive impairments of SAE. Enzyme‐linked immunosorbent assay (ELISA) was performed to determine 5‐HT levels in hippocampus, brainstem and frontal lobe of experimental groups.

**Results:**

Both LPS‐induced sepsis and CLP induced sepsis resulted in a notable learning deficit. Fluoxetine ameliorated, while cyproheptadine aggravated, cognitive impairment in two classic mouse models of SAE. The cognition‐enhancing effect of fluoxetine is reversed by WAY100635. Decreased 5‐HT levels in hippocampus, brainstem and frontal lobe were observed in LPS septic model and CLP septic model. Notably, both fluoxetine and cyproheptadine significantly increased 5‐HT levels in those brain regions in LPS septic model. Additionally, fluoxetine significantly increased 5‐HT levels in frontal lobe of CLP septic model.

**Conclusions:**

Our study demonstrated that serotonergic neurotransmission plays a significant role in mechanisms underlying cognitive impairment in SAE. These findings contribute to identification of novel targets to prevent and arrest cognitive impairment in SAE.

## Graphical abstract

Septic mice exhibited cognitive dysfunction and decreased 5‐HT in brain region associated with cognition, and cognitive performance was mediated by SSRI and 5‐HT receptor antagonist. Serotonergic neurotransmission plays a significant role in mechanisms underlying cognitive impairment in SAE.

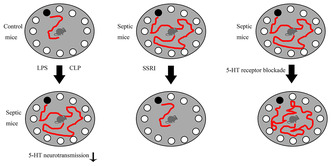

## 1 BACKGROUND

Sepsis‐associated encephalopathy (SAE) stands out as the most prevalent form of brain disorder observed in intensive care units worldwide.^1^ It is characterized by diffuse brain dysfunction that occurs as a result of sepsis, devoid of any direct central nervous system infection, structural abnormalities, or other types of encephalopathy.^1^ Remarkably, approximately 70% of sepsis patients develop SAE,^2^, ^3^ showcasing distinctive features such as cognitive dysfunction, memory loss, and abnormal behavior.^4^ Numerous studies have shown that SAE significantly heightens the risk of in‐hospital mortality and poor prognosis for sepsis patients.^5^ This, in turn, results in a notable 45% of sepsis survivors demonstrating long‐term cognitive impairment, leading to a significant decline in their quality of life.^6^ The underlying pathogenetic mechanisms of cognitive dysfunction of SAE have not been well established, and a limited number of effective clinical treatments are available. In this regard, there exists an urgent imperative to investigate the pathogenesis and potential therapeutic targets of SAE.

Previous studies have demonstrated alterations in serotonin level (5‐hydroxytryptamine [5‐HT]) in the brain of rodent models with sepsis.^7^, ^8^ The serotonergic neurotransmission is involved in diverse cognitive functions including memory.^9^ For instance, selective blockade of 5‐HT_6_ receptors could improve spatial recognition memory and reverse age‐related deficits in spatial recognition memory in the mouse.^10^ Additionally, SL65.0155, a serotonin 5‐HT_4_ receptor partial agonist, has a cognition‐enhancing effect in the model of amnesia.^11^ Serotonergic medications have also shown promise in reducing the risk of cognitive decline in Parkinson disease.^12^ Despite these insights, the specific role of serotonergic neurotransmission in SAE has not been characterized.

In this study, we hypothesized that abnormality of serotonergic neurotransmission might be associated with cognitive dysfunction in SAE. Our investigation initially commenced by examining the effect of serotonergic agents on cognitive performance of two classic mouse models of SAE, induced through lipopolysaccharide (LPS) injection and cecal ligation and puncture (CLP) respectively. Additionally, we explored the relationship between expression of 5‐HT in the cognition‐associated region of the brain and the cognitive function of SAE mice.

## 2 MATERIALS AND METHODS

### 2.1 Animals

Male wild‐type C57BL/6 mice (initial weight 18–22 g, 8–10 weeks old) were purchased from Hunan SJA Laboratory Animal Co., Ltd. (Changsha, China). Mice were accommodated in groups of up to five per cage under controlled conditions (18–25°C; 50%–60% humidity; 12/12 h light/dark cycle) and provided with rodent food and water ad libitum. Mice were handled for 2 min per day for 7 days before the establishment of the model of SAE took place. All animals were treated humanely. Study design and all animal experimental protocols were approved by the Ethics Committee of Xiangya Hospital, Central South University, Changsha, China (Protocol Number: 2023030178).

### 2.2 Pharmacology

Lipopolysaccharide (LPS) was purchased from Sigma‐Aldrich (Escherichia coli endotoxin 055:B5, catalog no. L2880, St. Louis, MO, USA). Fluoxetine (FLU, selective serotonin reuptake inhibitor [SSRI]) and cyproheptadine (CYP, nonselective 5‐HT2 receptor antagonist) were obtained from MeilunBio (Dalian, China).WAY100635 was obtained from MedChemExpress LLC (Monmouth Junction, NJ, USA). LPS, FLU, CYP and WAY100635 were all dissolved in 0.9% saline (vehicle), and administered via intraperitoneal (i.p.) injection.

### 2.3 Experimental design and drug administration

**FIGURE 1:**
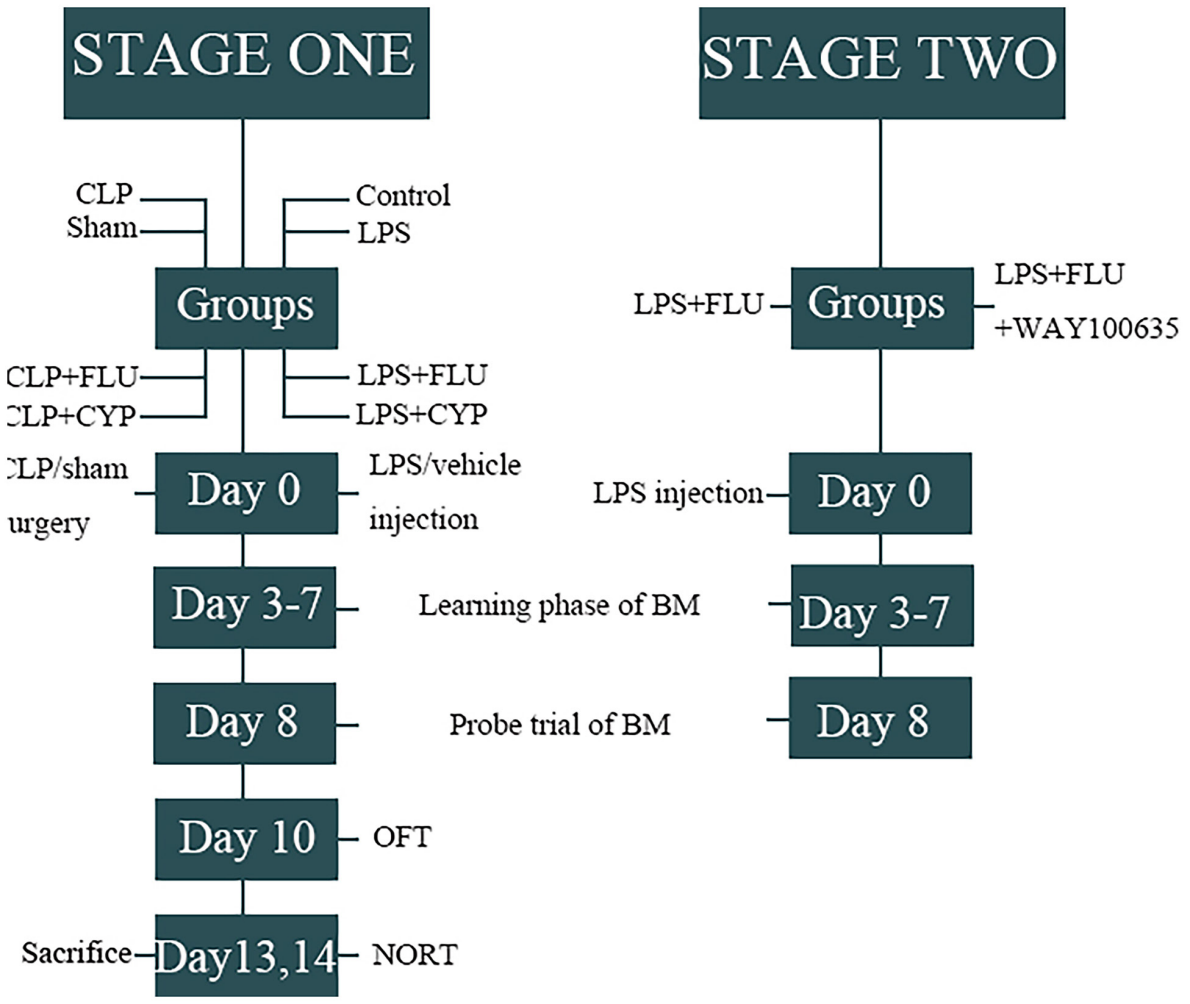
Schematic diagram of experiments. BM, barnes maze; CLP, cecal ligation and puncture; CYP, cyproheptadine; FLU, fluoxetine; LPS, lipopolysaccharide; NORT, new object recognition test; OFT, open field test.

The experimental design of the study is outlined in Figure 1. In this study, we randomly divided male mice into eight experimental groups: control, CLP, CLP + CYP, CLP + FLU (*n* = 8/group), sham, LPS + FLU + WAY100635 (*n* = 7/group), LPS (*n* = 16), LPS + CYP (*n* = 8), LPS + FLU (*n* = 23). The establishment of SAE models were executed through LPS injection and CLP, respectively. Fluoxetine (10, 20 mg/kg), cyproheptadine (20 mg/kg) and vehicle were administered intraperitoneally 30 min before trials. WAY100635 (1 mg/kg) was administered intraperitoneally 30 min prior to fluoxetine injection in LPS + FLU + WAY100635 group. The mice were anesthetized via isoflurane inhalation and killed by decapitation within 120 min after the last behavioral test, the brain tissue was collected and frozen for histological examination. This study involved a total of 115 mice. The survival rates on the day 14 were 100% in both the control and sham group. The survival rates of mice within 3 days after receiving CLP or LPS injection were 87.9% (58/66) and 82.4% (28/34), respectively. Subsequently, a total of 86 septic mice entered the behavioral experiment phase. Among them, the survival rates of LPS mice receiving or not receiving serotonergic agents were 90.5% (38/42) and 100% (16/16), respectively. Additionally, the survival rates of CLP mice receiving or not receiving serotonergic agents were 88.9% (16/18) and 80% (8/10), respectively.

### 2.4 Establishment of SAE model

Mice in the control group received normal saline. To establish the LPS‐induced mouse sepsis model of SAE, mice were injected intraperitoneally with LPS (10 mg/kg). For the CLP‐induced SAE model, mice underwent a procedure previously described.^13^ Briefly, mice were anesthetized with isoflurane, followed by a ventral midline incision (1 cm). The cecum was isolated, ligated with 3.0 silk sutures (10% ligation), pierced twice with a 21‐gauge needle, and squeezed lightly to force intestinal content into the peritoneal activity before closing the incision. Finally, the cecum was gently returned to the abdominal cavity, and the abdomen was sutured. Following the surgery, mice were resuscitated with 1 mL of normal saline. Mice in the sham group underwent all surgical procedures, but the cecum was neither ligated nor perforated.

### 2.5 Behavioral tests

Behavioral tests began 72 h after the LPS infusion or CLP. All behavioral experiments were conducted between 09:00 and 17:00. Behaviors were recorded and analyzed. The schedule of behavioral tests is shown in Figure 1.

#### 2.5.1 Open field test

To measure the locomotor activity, the open field test (OFT) was performed after LPS injection, utilizing an open field plastic chamber (40 × 40 × 50 cm). To start the trial, mice were placed into the center of the open field and allowed to move freely for 10 min. For each trial, the open field chamber was thoroughly cleaned with 70% ethanol solution and afterward by dry paper towels. The total distance traveled and velocity were calculated.

#### 2.5.2 Barnes maze

The Barnes maze (BM) was designed for testing spatial learning and memory. BM consisted of a circular table (92 cm diameter, 105 cm high) with 20 holes (19 empty holes and 1 target hole with escape box) distributed around the perimeter. The mice were placed in a cylindrical container at the center of the table for 5 s before each trial. During the learning phase, mice were allowed to freely explore the target hole for 3 min per trial (3 trials per day) for 5 consecutive days. If a mouse was unable to locate the target hole, it was guided towards that location and placed in the escape box for 60 s. Trials were separated by 30–45 min. During the memory phase, the escape box was removed, allowing mice to move freely on the table for 3 min. After each trial, the arena was cleaned with 75% alcohol to prevent odor cues, and the maze was rotated to eliminate the use of intramaze cues. The time taken for the first encounter with the escape box was termed primary latency and success rate were defined as the proportion of trials in which mice reached the target hole within 3 min. During the learning phase, primary latency, success rate was recorded and analyzed. During the memory trial, primary latency to the target hole, time spent in the target quadrant, mean velocity was recorded and analyzed.

#### 2.5.3 New object recognition test

The New object recognition test (NORT) was used to assess recognition memory in the subject mice. Mice were placed in an empty box (35 × 35 × 20 cm) for 10 min to facilitate habituation 24 h prior to acquisition trial. During the acquisition trial, mice were allowed to freely explore two identical objects for 5 min and then returned to their home cage. After a 24 h break, a 5 min retention trial was performed to evaluate long‐term memory in which one of the two familiar objects was replaced by a novel one. The percentage of time spent exploring the new was calculated. To minimize odor cues, the arenas and objects were carefully cleaned with 70% alcohol between the trials.

### 2.6 Enzyme‐linked immunosorbent assay

Tissues from hippocampus, brainstem and frontal lobe were collected for the detection of 5‐HT concentrations using a competitive enzyme‐linked immunosorbent assay (Uscn Life Science, Inc., Wuhan, China). All tests were performed according to the manufacturer's instructions. Each sample was tested in duplicate.

### 2.7 Statistical analyses

Data were expressed as the mean and standard deviation. The Shapiro–Wilk test and Kolmogorov Smirnov test were used to evaluate the normal distribution of the data, while the Levene test was employed to compare variances before applying a parametric test to compare the differences between groups. If data did not fulfill the requirements of a parametric test, a nonparametric test would be selected. The groups were compared using two‐sample *t*‐test for parametric data and Mann Whitney *U* test for non‐parametric data. The qualitative variables were compared using Pearson's *X* ^2^ or Fisher exact test, as appropriate. Time series data were analyzed with repeated measures two‐way ANOVA. Kruskal‐Wallis test followed by Bonferroni Procedure (non‐parametric) was used to account for multiple comparisons. The results were considered statistically significant when *p* < 0.05. All statistical analyses were two‐sided and were conducted using the Statistical Package for the Social Sciences version 23.0. GraphPad Prism 8 was used to make graphs.

## 3 RESULTS

### 3.1 Fluoxetine improved cognitive dysfunction of LPS‐induced septic mice

**FIGURE 2:**
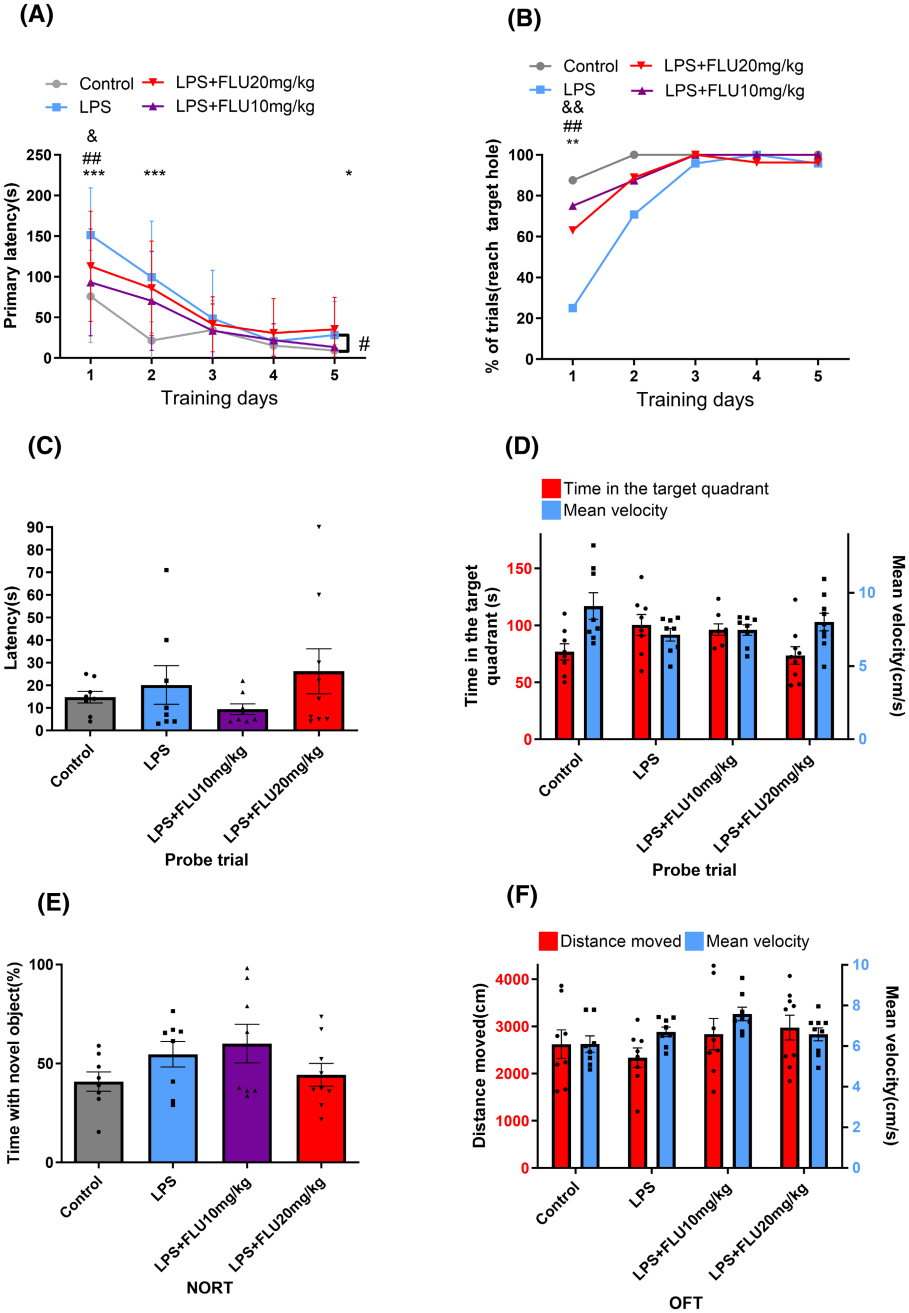
Administration of fluoxetine rescues LPS‐induced learning deficit. (A, B) BM data show that LPS group showed spatial learning deficit in learning phase; administration of fluoxetine improved LPS‐induced learning dysfunction; repeated‐measurements ANOVA found treatment with 10 mg/kg of fluoxetine shorten the primary latency of LPS‐induced septic mice (A). (C, D) During the probe trial of BM, the latency, time spent in the target quadrant and mean velocity were not affected. (E) No difference was seen between groups in NORT (Control vs. LPS; LPS + FLU vs. LPS). (F) The locomotor activity was not affected in OFT (Control vs. LPS; LPS + FLU vs. LPS). *Control versus LPS; ^#^LPS versus LPS + FLU (10 mg/kg); ^&^LPS versus LPS + FLU (20 mg/kg). *p < 0.05, **p < 0.01, ***p < 0.001, ^#^ *p* < 0.05, ^##^ *p* < 0.01, ^&^ *p* < 0.05, ^&&^ *p* < 0.01.

BM was used to test spatial learning and memory. In comparison to the control group, the LPS group exhibited significantly longer primary latency on days 1, 2, 5 (*p* < 0.001 for days 1, 2, *p* = 0.014 for day 5; Figure 2A) and lower success rate on day 1 (*p* = 0.001; Figure 2B). During the memory test (probe trial) on day 6, wherein the escape box was removed, no difference in latency, time spent in the target quadrant and mean velocity between LPS group and control group (*p* > 0.05 for all; Figure 2C,D). These findings suggest that LPS‐induced sepsis induces spatial learning impairment in our study.

The effect of fluoxetine on cognitive dysfunction was examined. During the learning phase in the BM, the LPS + FLU group demonstrated shorter primary latency (*p* = 0.002 and 0.015 for FLU at 10, 20 mg/kg, respectively) and higher success rate (*p* = 0.001 and 0.007 for FLU at 10, 20 mg/kg, respectively) on learning day 1 compared to the mice in LPS group (Figure 2A,B), and repeated‐measurements ANOVA revealed that treatment with 10 mg/kg of fluoxetine shorten the primary latency of LPS‐induced septic mice (*p* = 0.005, *F* = 9.46; Figure 2A). However, no effect of fluoxetine on memory and locomotor ability was detected in the probe trial (*p* > 0.05 for all; Figure 2C,D).

We administered the NORT to assess long‐term memory. There were no discernible differences seen between groups in the time spent on exploring new objects (*p* > 0.05; Figure 2E). Additionally, the OFT was used to assess the locomotor activity, revealing no significant differences in the distance moved and mean velocity between control group and LPS group, and the same was found for the LPS + FLU group compared to the LPS group (*p* > 0.05 for both; Figure 2F), which means administration of fluoxetine (10 and 20 mg/kg) had no significant impact on locomotor activity of LPS‐induced septic mice.

### 3.2 Cyproheptadine aggravated cognitive dysfunction of LPS‐induced septic mice

**FIGURE 3:**
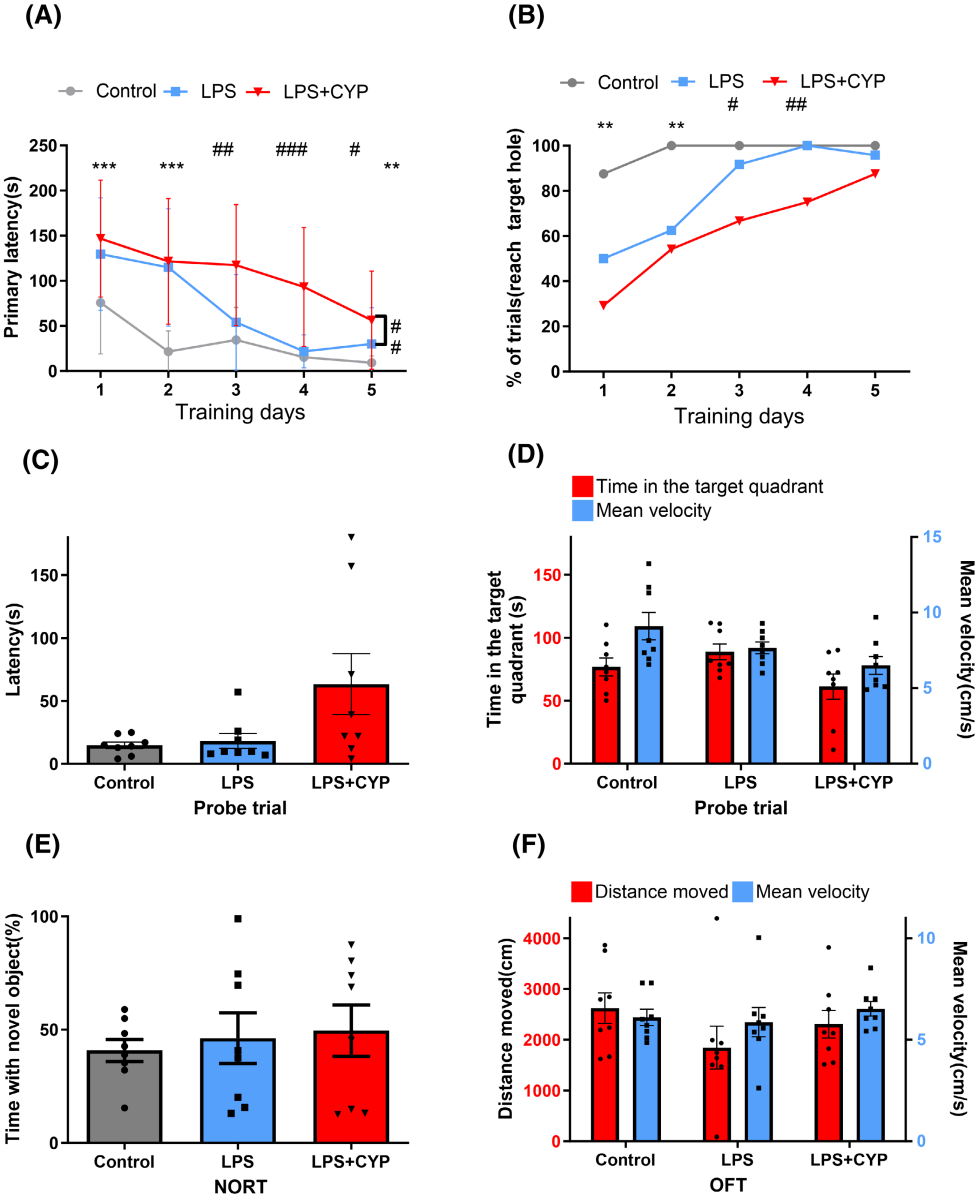
Administration of cyproheptadine aggravated LPS‐induced learning deficit. (A, B) BM data show that LPS group showed spatial learning deficit in learning phase; administration of cyproheptadine aggravated LPS‐induced learning dysfunction; repeated‐measurements ANOVA found the primary latency of LPS‐induced septic mice was prolonged by treatment with 20 mg/kg of cyproheptadine (A). (C, D) During the probe trial of BM, the latency, time spent in the target quadrant and mean velocity were not affected. (E) No difference was seen between groups in NORT (Control vs. LPS; LPS + CYP vs. LPS). (F) The locomotor activity was not affected in OFT. *Control versus LPS. ^#^LPS versus LPS + CYP. *p < 0.05, **p < 0.01, ***p < 0.001, ^#^ *p* < 0.05, ^##^ *p* < 0.01, ^###^ *p* < 0.001.

In order to further investigate the role of serotonergic neurotransmission in SAE, we conducted a study examining the effect of cyproheptadine (20 mg/kg, nonselective 5‐HT_2_ receptor antagonist) on cognitive function of LPS‐induced septic mice. In BM, LPS group exhibited a longer primary latency on learning days 1, 2, 5 (*p* < 0.001 for day 2, *p* = 0.003 for days 1, 5; Figure 3A) and lower success rate on learning days 1, 2 than control group (*p* = 0.005 and 0.003, respectively; Figure 3B). It is worth mentioning that the preinjection with cyproheptadine induced longer primary latency (*p* < 0.001 for day 4, *p* = 0.004 and 0.013 for days 3, 5) and lower success rate during learning phase (*p* = 0.033 and 0.002 for days 3, 4; Figure 3A,B). The repeated‐measurements ANOVA revealed that cyproheptadine prolonged the primary latency of LPS‐induced septic mice (*p* = 0.001, *F* = 13.41; Figure 3A).Cyproheptadine showed no effect on cognitive performance and locomotor function during memory phase (*p* > 0.05 for both; Figure 3C,D). In NORT, no differences were observed between groups in the time spent on exploring new object (*p* > 0.05; Figure 3E). The distance moved and mean velocity was not significantly different between the groups in OFT (*p* > 0.05 for both; Figure 3F), indicating that administration of cyproheptadine had no effect on locomotor activity of LPS‐treated mice.

### 3.3 The CLP‐induced cognitive dysfunction was mediated by serotonergic agents

In addition to LPS injection, CLP is another classic method to establish animal models of sepsis. To determine if the mediating effect of serotonergic agents on cognitive performance and 5‐HT level of the brain in septic mice is dependent on sepsis‐induction methods, we also examined the role of serotonergic neurotransmission in CLP‐induced septic mice.

**FIGURE 4:**
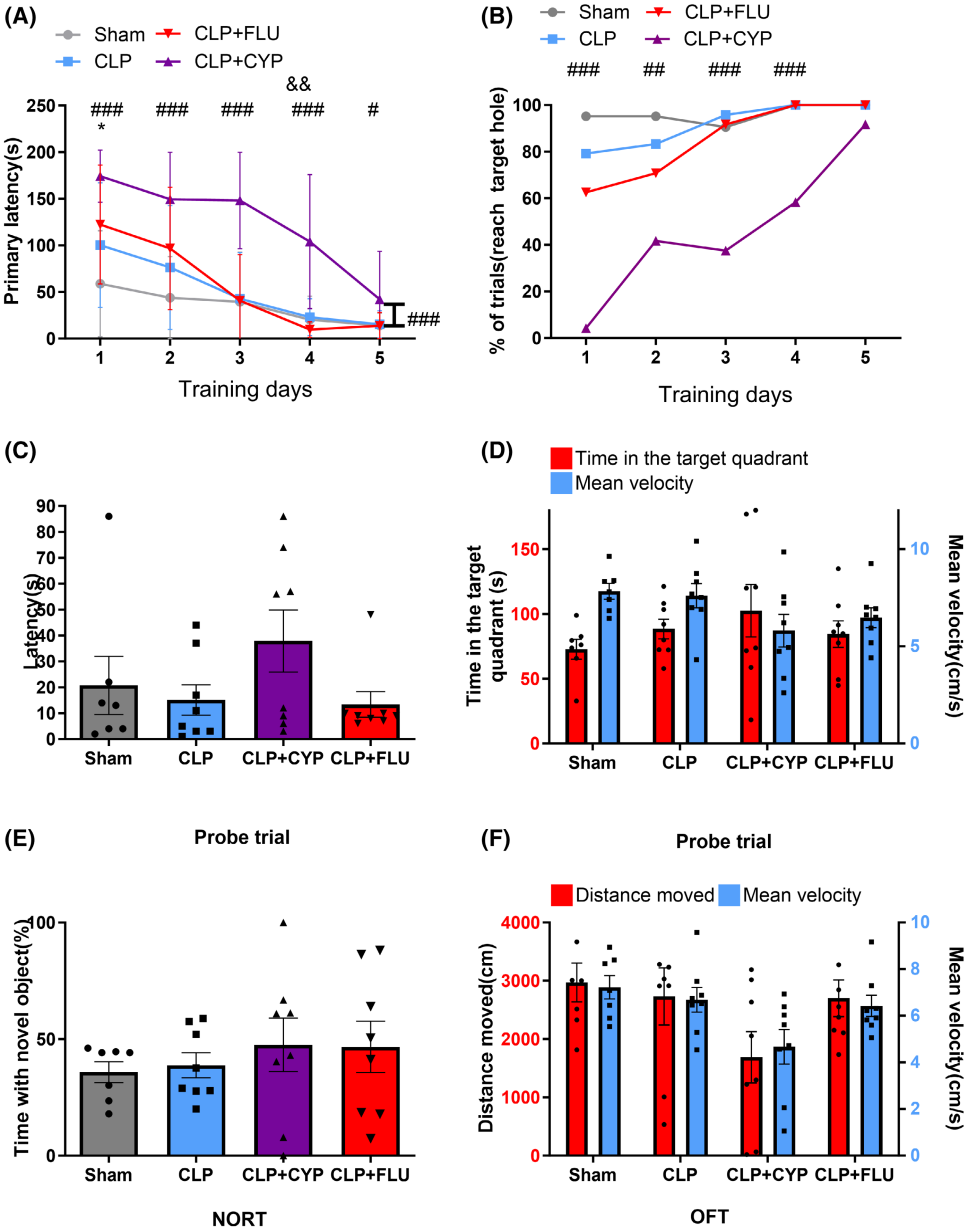
The CLP‐induced cognitive dysfunction was mediated by serotonergic agents. (A, B) BM data show that CLP group showed longer primary latency in learning day 1 compared with Sham group; fluoxetine shorten primary latency of CLP mice in learning day 4 (CLP vs. CLP + FLU); cyproheptadine prolonged primary latency and decreased the success rate of CLP mice during learning phase (CLP vs. CLP + CYP); repeated‐measurements ANOVA found the primary latency of CLP mice was prolonged by cyproheptadine (A). (C, D) During the probe trial of BM, the latency, time spent in the target quadrant and mean velocity were not affected. (E) No difference was seen between groups in NORT (Sham vs. CLP; CLP vs. CLP + CYP; CLP vs. CLP + FLU). (F) The locomotor activity was not affected in OFT (Control vs. LPS; LPS + FLU vs. LPS). *Sham versus CLP; ^#^CLP versus CLP + CYP; ^&^CLP versus CLP + FLU. *p < 0.05, ^#^ *p* < 0.05, ^##^ *p* < 0.01, ^###^ *p* < 0.001, ^&&^ *p* < 0.01.

As compared to the sham group, the CLP group displayed significantly longer primary latency on day 1 (*p* = 0.035; Figure 4A) in the learning phase of BM. The mice in the CLP + CYP group exhibited significantly poorer primary latency (*p* < 0.001 for days 1–4, *p* = 0.018 for day 5; Figure 4A) and success rate than those in the CLP group (*p* < 0.001 for days 1, 3, 4; *p* = 0.003 for day 2; Figure 4B). The repeated‐measurements ANOVA revealed that cyproheptadine prolonged the primary latency of CLP mice (*p* < 0.001, *F* = 70.04; Figure 4A).The CLP + FLU group also exhibited shorter primary latency on learning day 4 than CLP mice (*p* = 0.008; Figure 4A). During the probe trial of BM, there was no difference in latency, time spent in the target quadrant and mean velocity between groups (*p* > 0.05 for all; Figure 4C,D). In NORT, there was no difference seen between groups (*p* > 0.05; Figure 4E). In OFT, there was no difference in the distance moved and mean velocity between CLP group and Sham group, and serotonergic agents produced no significant effect on locomotor activity of CLP mice (*p* > 0.05 for all, Figure 4F).

### 3.4 5‐HT concentrations in brain of mouse model of SAE

**FIGURE 5:**
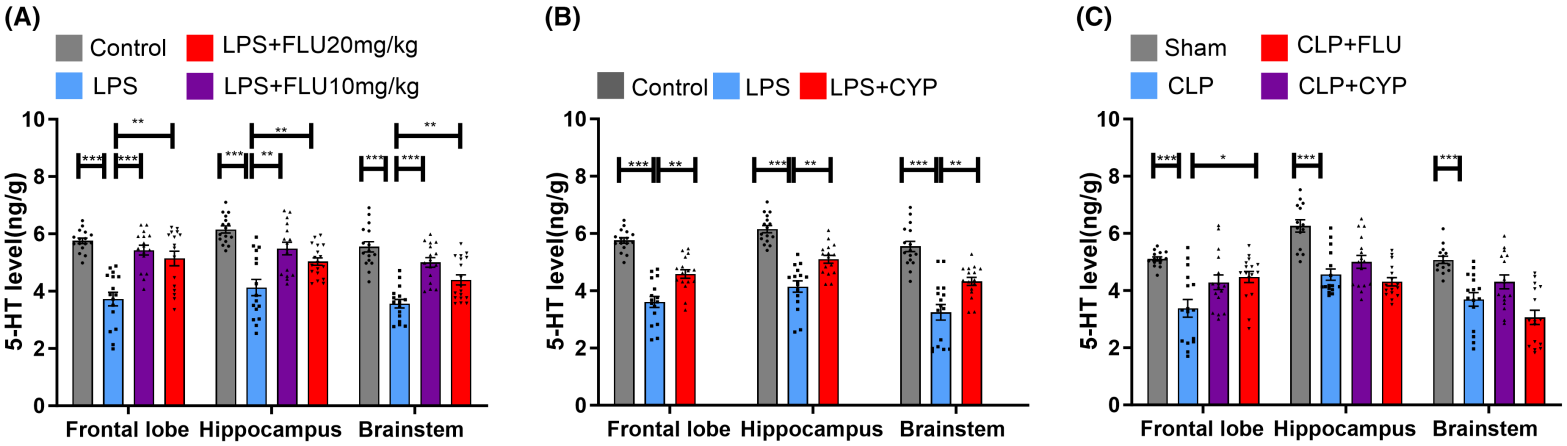
5‐HT concentrations in brain of mouse model of SAE. (A) In the LPS model, the LPS group showed significantly lower 5‐HT levels in brainstem, hippocampus and frontal lobe, compared with the control group; treatment of fluoxetine significantly increased 5‐HT levels in brainstem, hippocampus and frontal lobe. (B) In the LPS model, treatment of cyproheptadine significantly increased 5‐HT levels in brainstem, hippocampus and frontal lobe. (C) In the CLP model, the CLP group showed significantly lower 5‐HT levels in brainstem, hippocampus and frontal lobe, compared with the sham group; treatment of fluoxetine significantly increased 5‐HT level in frontal lobe. *p < 0.05, **p < 0.01, ***p < 0.001.

To further explore the molecular mechanisms through which the serotonergic neurotransmission mediated cognitive dysfunction in the mouse model of SAE, we used ELISA to examine the levels of 5‐HT in brainstem, hippocampus and frontal lobe. In the LPS model, the LPS group showed significantly lower 5‐HT levels in brainstem, hippocampus and frontal lobe, compared with the control group (*p* < 0.001 for all; Figure 5A,B). Fluoxetine significantly increased 5‐HT levels in those regions (10 mg/kg: *p* < 0.001 for frontal lobe and brainstem, *p* = 0.002 for hippocampus; 20 mg/kg: *p* = 0.001 for frontal lobe, *p* = 0.003 for brainstem, *p* = 0.018 for hippocampus; Figure 5A). Cyproheptadine significantly increased 5‐HT levels in brainstem, hippocampus and frontal lobe (*p* = 0.005, 0.001, 0.002, respectively; Figure 5B). In the CLP model, the CLP group displayed significantly lower 5‐HT levels in brainstem, hippocampus and frontal lobe compared to the sham group (*p* < 0.001 for all; Figure 5C). Fluoxetine significantly increased 5‐HT level in frontal lobe (*p* = 0.012; Figure 5C), and cyproheptadine produced no significant effect on 5‐HT levels in brainstem, hippocampus and frontal lobe of CLP‐induced septic mice (*p* > 0.05 for all; Figure 5C).

### 3.5 The cognition‐enhancing effect of fluoxetine is reversed by a 5‐HT_1A_ receptor antagonist

**FIGURE 6:**
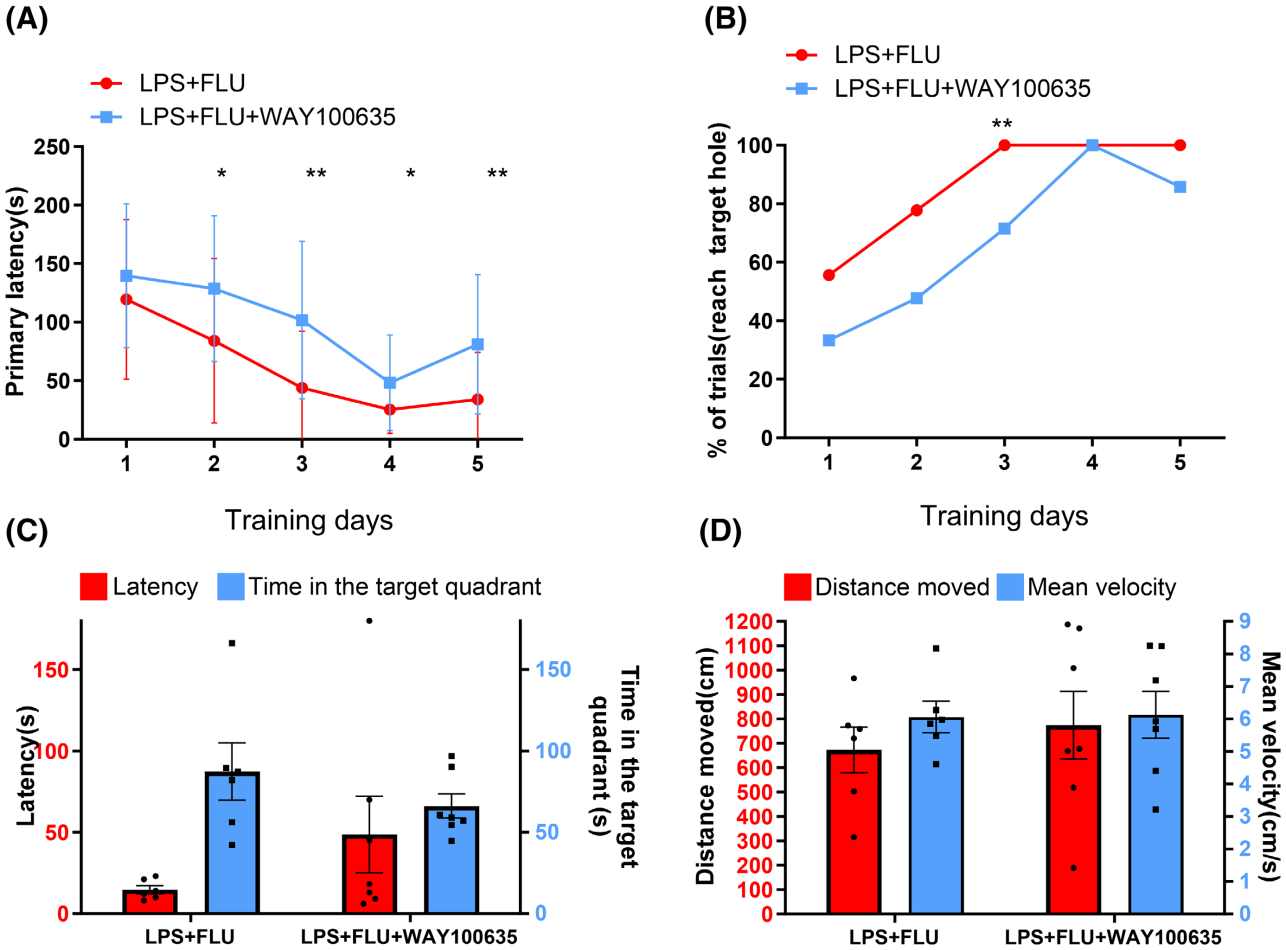
The cognition‐enhancing effect of fluoxetine is reversed by a 5‐HT_1A_ receptor antagonist. (A, B) Compared with the primary latency in LPS + FLU group, administration of the 5‐HT_1A_ receptor antagonist WAY100635 (1 mg/kg) 30 min prior to fluoxetine injection induced significantly longer primary latency on days 2–5 and lower success rate on day 3 during learning phase. (C, D) No difference in the latency, time spent in the target quadrant, mean velocity and distance moved during probe trial between groups. *p < 0.05, **p < 0.01.

To further investigate the mechanism through which fluoxetine improved cognitive impairment, the 5‐HT receptor antagonists were administered prior to fluoxetine injection. In comparison to the primary latency in the presence of 20 mg/kg fluoxetine alone, administration of WAY100635 (1 mg/kg) 30 min prior to fluoxetine injection induced significantly longer primary latency on days 2–5 (*p* = 0.029, 0.006, 0.045, 0.004, respectively; Figure 6A) and lower success rate on day 3 during learning phase (*p* = 0.004, Figure 6B). No difference in cognitive performance and locomotor activity during probe trial between groups (*p* > 0.05 for all; Figure 6C,D).

## 4 DISCUSSION

Previous studies have indicated that the 5‐HT neurotransmission plays a crucial role in regulating various physiological functions, including cognition, sleep, and emotions. The expression of 5‐HT receptors has been observed in brain regions associated with learning and memory, such as the cortex, amygdala, and hippocampus.^14^ Abnormalities in the serotonergic neurotransmission can lead to impaired memory and altered synaptic plasticity in the hippocampus.^15^ For instance, the depletion of 5‐HT in rats has been shown to result in deficits in object recognition and memory.^16^ Rats injected with a 5‐HT_1A_ receptor agonist (8‐OH‐DPAT), either bilaterally into the hippocampus or into the lateral ventricle, also display spatial memory deficits.^17^ Moreover, it has been found that the severity of cognitive impairment in AD patients is positively correlated with the density of 5‐HT_1A_ receptors in the frontal lobe, while being negatively correlated with the levels of 5‐HT in the frontal lobe.^18^ Furthermore, the use of mianserin (an antagonist of the 5‐HT_2A_ receptor) has been shown to improve learning and memory function in patients with schizophrenia.^19^ These findings highlight the intricate involvement of serotonergic neurotransmission in the mechanisms underlying cognitive dysfunction in central nervous system disorders.

The mortality rate associated with sepsis continues to pose a significant global public health challenge. However, the mechanisms by which sepsis affects cognitive function are unclear. Previous studies did not pay sufficient attention to the relationship between the serotonergic neurotransmission and SAE. Therefore, this experiment fills a critical gap in our understanding.

The present study shows that administration of fluoxetine, a selective serotonin reuptake inhibitor, is followed by enhanced cognitive capacity in the mouse model of SAE. Moreover, septic mice showed a decreased level of 5‐HT in hippocampus, brainstem and frontal lobe compared with control mice. Our findings uncover an innovative mechanism whereby the serotonergic neurotransmission mediated cognitive deficits of SAE.

How does fluoxetine work to improve cognitive performance in septic mice? Our findings demonstrated that the effect of fluoxetine on cognition was prevented by the 5‐HT_1A_ receptor antagonist WAY100635. These findings indicate that fluoxetine alleviates cognitive deficit by enhancing serotonergic neurotransmission and activating 5‐HT_1A_ receptors. Fluoxetine alleviates cognitive deficit in SAE likely through the activation of postsynaptic 5‐HT_1A_ receptors, as presynaptic 5‐HT_1A_ receptors are autoreceptors, and activation of these receptors would inhibit 5‐HT release.^20^ Moreover, the deteriorated cognitive performance of septic mice was induced by cyproheptadine, which implies 5‐HT_2_ be involved in mechanism of cognitive impairments in SAE, thereby yielding fresh perspectives on the pathogenesis of SAE and potential therapeutic targets that are of great significance and innovation.

In previous investigations, behavioral alteration in BM, NORT, morris water maze, and similar tests could be observed in CLP and LPS models of SAE.^21^ In the present study, both the LPS and CLP models exhibited impaired spatial learning in BM. However, no cognitive deficits observed in the probe trial of BM and NORT, aligning partially with previous studies. Potential factors contributing to these discrepancies include variations in LPS dosage, length of ligated cecum, mouse strain, age, interval between the model induction and test, experimental environment, as well as the timing of training and test. These factors collectively influence both the extent of cognitive dysfunction and the results of behavioral tests.^21^

There are few studies on the central 5‐HT levels in sepsis models. A study by Mota et al.^8^ found a decrease in 5‐HT levels in the anteroventral region of the hypothalamus in rats 5 h after receiving an intravenous injection of LPS. In contrast, the study by Shimizu,^7^ which reported an increase in 5‐HT levels in the cerebral cortex, hippocampus, and striatum of male wistar rats 48 h after undergoing CLP, contradicts the findings of our study, timing of sample collection may account for the discrepancy in hippocampal 5‐HT level.

Both fluoxetine and cyproheptadine significantly increased the levels of 5‐HT in the hippocampus, brainstem, and frontal lobe in the LPS model. Conversely, in the CLP model, fluoxetine significantly elevated 5‐HT levels in the frontal lobe, while cyproheptadine induced a trend of increased 5‐HT level in the frontal lobe. This difference may be attributed to the heterogeneity of the models. Cyproheptadine, with its high affinity for 5‐HT2 receptors,^22^, ^23^, ^24^ provides potential insights into its mechanism of action. Previous studies found the selective 5‐HT_2C_ receptor antagonists SB 242084 and RS 102221 have been shown to replicate the comparable enhancement of SSRI‐induced increases in serotonin levels in the hippocampus. Moreover, ketanserin, a non‐selective 5‐HT_2_ receptor antagonist, exhibited a strong augmentation of SSRI‐induced elevations in 5‐HT level in both hippocampal and cortical microdialysis in rats.^25^ These findings provide potential insights into the mechanism by which cyproheptadine increases the concentration of 5‐HT in the brain.

The observed increase in 5‐HT levels in the brain region following cyproheptadine administration was accompanied by a deterioration in cognitive behavior in septic mice. This effect is likely attributed to the antagonism of the 5‐HT_2_ receptors. Key brain regions such as the cortex, hippocampus, basal ganglia, and forebrain, are primarily associated with cognitive function. Notably, the expression of the 5‐HT_2A_ receptors in the central nervous system is particularly extensive in those regions.^26^ Systemic activation of 5‐HT_2A_ receptors with the selective agonist, TCB‐2 significantly enhanced the consolidation of object memory in NORT, and enhancing effect of TCB‐2 could be blocked by pretreatment with the 5‐HT_2A_ receptor antagonist, MDL 11939, which suggests that 5‐HT_2A_ receptors activation potentiates memory consolidation.^27^ Additionally, the relevance of the 5‐HT_2A_ receptors for cognition was also demonstrated by results of a study showing that systemic administration of 5‐HT_2A_ receptor antagonist (M100907) significantly impaired spatial reversal learning of rats.^28^ Therefore, the potential cognitive improvement induced by the elevation of 5‐HT level may be outweighed by deleterious effects resulting from the nonselective antagonism of 5‐HT2 receptors in our study.

In contrast, Amodeo et al.^29^ found both systematic administration of risperidone (5‐HT_2A_ receptor antagonist) and MDL 11939 improved reversal learning in the mouse model of autism spectrum disorder. In addition, Naghdi et al.^30^ found microinfusion of ritanserin (5HT_2A/2C_ receptor antagonist) into the CA1 region of the hippocampus improves performance of rats in spatial discrimination task of morris water maze. Thus, it would appear that the cognition‐modifying effect of the 5‐HT receptors depends on the tested task and cognition system, affected brain regions and subtype of receptors, as well as pathologic condition.

The CLP and LPS models of sepsis are the most frequently used to assess cognitive dysfunction in the studies of SAE.^21^ Moreover, CLP is widely regarded as the gold standard model for sepsis research due to its closely resembles the progression and characteristics of human sepsis.^31^ In present study, the role of serotonergic neurotransmission in the mechanisms of cognitive dysfunction has been confirmed in both CLP and LPS models of SAE, indicating the generality of our conclusions.

The dosages of LPS vary among studies investigating the animal model of SAE. Some studies have employed LPS dosages ranging from 2 to 20 mg/kg.^32^, ^33^, ^34^, ^35^ In our study, we selected a dose of 10 mg/kg LPS (derived from Escherichia coli serotype 055:B5), as our preliminary experiments revealed no significant difference in cognitive function were observed between mice receiving 5 mg/kg LPS and healthy control mice. In contrast, a recent study demonstrated noticeable cognitive impairments in mice receiving 2 mg/kg LPS (derived from Escherichia coli serotype 0111:B4).^35^ The discrepancy in effective dosages of LPS may be attributed to several potential factors. Firstly, variations in toxicity could arise from different production batches and distinct LPS subtypes employed in the studies. Secondly, differences in the behavioral testing protocols and the timing of their implementation across various investigations may also contribute to the observed variations in cognitive performance.

In the current study, we only focused on the role of serotonergic neurotransmission on cognitive impairment of the mouse model of SAE by systemic administration of various serotonergic agents. More studies are needed in the future to investigate the detailed action such as the molecular mechanism underlying effects of serotonergic neurotransmission on cognitive impairment and the nuclei and receptors where 5‐HT exerts its effect in the brain. These endeavors hold promise in refining forthcoming therapeutic strategies aimed at mitigating cognitive impairment in patients with SAE.

## 5 CONCLUSIONS

In summary, our data demonstrate that fluoxetine and cyproheptadine respectively ameliorated and aggravated cognitive impairment in two classic mouse models of SAE. Furthermore, the cognition‐enhancing effect of fluoxetine can be blocked by antagonism of 5‐HT_1A_ receptors. In addition, the alteration of 5‐HT concentration was observed in mouse models of SAE. These findings suggest that serotonergic neurotransmission plays a significant role in mechanisms underlying cognitive impairment in SAE. Additionally, our findings indicated that 5‐HT_1A_ and 5‐H_A2_ receptors play a role in the mediating of cognition in SAE through serotonergic neurotransmission.

## AUTHOR CONTRIBUTIONS

ZT, FT: conceptualization. ZT, CZ: methodology. CZ, ZT: investigation. CZ, JL: formal analysis.CZ: resources, writing, and original draft. LZ, JP, XW and JL: writing‐review. ZT: editing and Supervision. All authors have read and approved the manuscript.

## FUNDING INFORMATION

This study was funded by the discipline development fund of Xiangya Hospital. The funders had no role in the design of the study and collection, analysis, and interpretation of data and in writing the manuscript.

## CONFLICT OF INTEREST STATEMENT

The authors declare no conflicts of interest.

## Data Availability

The datasets used and/or analyzed during the current study are available from the corresponding author on reasonable request.
